# Hiding Within the Cracks: Case Report of Rare Sutural Bone Found at the Nasion

**DOI:** 10.7759/cureus.1333

**Published:** 2017-06-10

**Authors:** Bryan Edwards, Joy MH Wang, Joe Iwanaga, Jennifer Luviano, Marios Loukas, Rod J Oskouian, R. Shane Tubbs

**Affiliations:** 1 Department of Anatomical Sciences, St. George's University School of Medicine, Grenada, West Indies; 2 Department of Anatomical Sciences, St. George's University School of Medicine, Grenada, West Indies; 3 Seattle Science Foundation; 4 Neurosurgery and Behavior, The Allen Institute for Brain Science; 5 Neurosurgery, Complex Spine, Swedish Neuroscience Institute; 6 Neurosurgery, Seattle Science Foundation

**Keywords:** skull, skull closure, cranium, nasion, suture, imaging, anatomy, wormian bone

## Abstract

Pathology such as skull fractures can be misdiagnosed in the presence of anatomical variations. One variant that has had little description in the literature are the sutural bones associated with the nasal bones. Herein, we describe a case of a rare sutural bone at the nasion, between the bones of the right nasal, frontal, and maxillary frontal process. To our knowledge, this is the first report of such a variant bone in this location, and such it should be considered by clinicians when evaluating patients for pathology in this region.

## Introduction

Arising from separate ossification centers during development, sutural bones, also known as wormian bones, are anatomical variants defined as islands of compact bone within sutures between skull bones and have been found in healthy children without a history of cranial trauma or underlying connective tissue disorders. The prevalence of wormian bones within the general population is variable, with reports ranging from 8% to 15% [1]. The rates are consistently higher in those with underlying medical conditions such as hypothyroidism [2], osteogenesis imperfecta [3], and cleidocranial syndrome [4].

Independent of subject type or health status, the most common location for these accessory bones is within the lambdoid suture. Other reported sites include the asterion, pterion, sagittal suture, parietotemporal suture, occipitomastoid suture, and coronal suture [1]. Here, we report an accessory suture bone found at the nasion, an extremely rare finding. We also review the function of sutures and their role in cranial bone development, discuss the presence of sutural bones in skull deformities and underlying diseases, hypothesize possible etiologies, and suggest areas of further study.

## Case presentation

**Figure 1:**
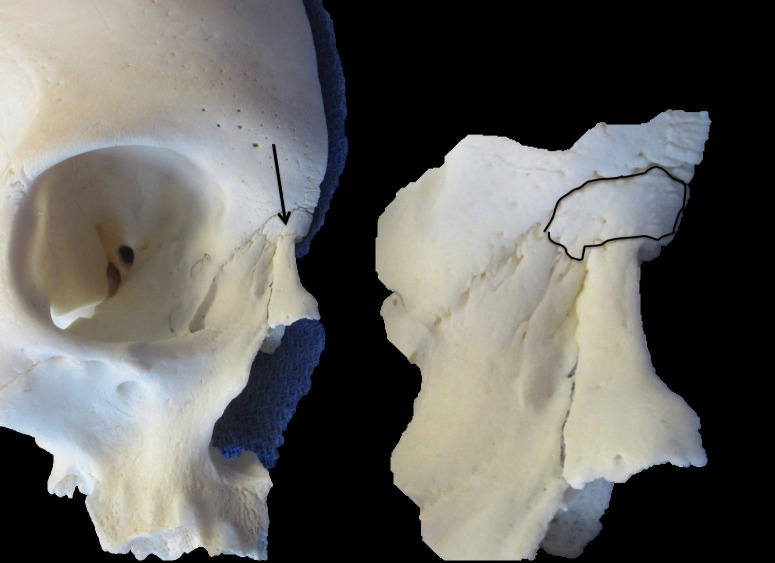
Skull specimen A. Anterior view noting the rare wormian bone (arrow) lodged between the frontal, nasal, and maxillary bones. B. Zoomed in view of the outlined bone shown in A.

During a routine examination of a skull specimen derived from an adult female, a very unusual bone (Figure 1) was identified at the nasion. Specifically, the 1 cm (length) x 0.5 cm (width) bone was interposed between the bones of the right nasal, frontal, and maxillary frontal process. Internally, the bone rested just inferior to the right frontal sinus but did not reach posteriorly to the ethmoid bone. All sutures of the calvaria were closed, and there were no signs of craniosynostosis or major asymmetries of the skull. However, the overall size of the skull was small at 16 cm (anteroposterior length) x 13 cm (height). No other osteological variations were noted, other than a small (4 x 5 mm) right-sided pterional sutural bone and a left-sided small (4 x 5 mm) sutural bone in the lateral most part of the lambdoidal suture at the asterion.

## Discussion

**Figure 2:**
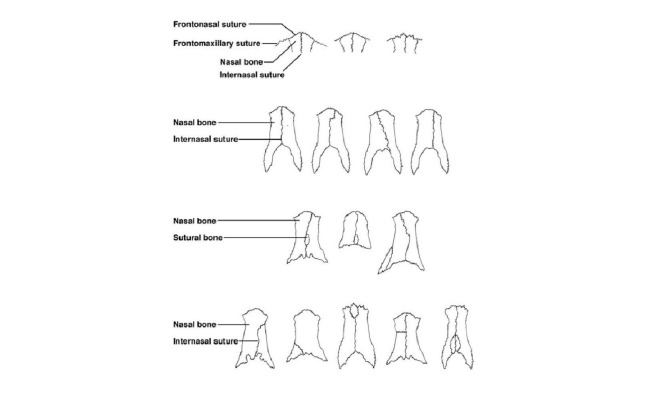
Schematic drawing of variants of the sutures and sutural bones previously described in the region of the nasal bones.

The intranasal suture, like all sutures, is a fibrous joint, giving flexibility to the bones they articulate with (Figure 2). Sutures allow the cranium to contort and squeeze through the vaginal canal during birth, to expand and accommodate our growing brains throughout infancy and childhood, and to absorb mechanical stress, providing greater resilience against injury [5]. Additionally, sutures play an important role in the osteogenesis of expanding cranial bones. During gestation, the ossification centers of the cranial flat bones proliferate and grow toward each other.  As they reach a location and width at which their edges create distinct areas, we call them sutures. As the expanding brain pushes skull bones outward, sutures are stretched progressively wider until an osteogenic response is triggered, causing osteogenic cells within the suture to mineralize the edges of the bone in their attempt to maintain a fixed suture width [5]. Parr et al. [6] demonstrated this osteogenic process by mechanically separating the intranasal suture and then measuring the resulting amount of new bone growth along the bone-suture interface.  

Given that sutures have demonstrated osteogenic capabilities at the bone suture-interface when placed under strain, it seems plausible that distinct bone growth within the suture may also occur under the same stimulus. The results of studies examining skulls from various cultures who practice intentional head-deforming rituals corroborate this hypothesis. White [7] examined the ancient adult skulls of a Mayan group in the Lamania region who practiced frontal-occipital skull binding rituals on all offspring from birth until one year of age, resulting in brachycephaly in a majority of their population. Out of the 16 deformed skulls studied, 88% had wormian bones. Sanchez et al. demonstrated a similar correlation in their study of deformed skulls from Chichen Itza, Mexico, and Ancon, Peru [8].

**Figure 3:**
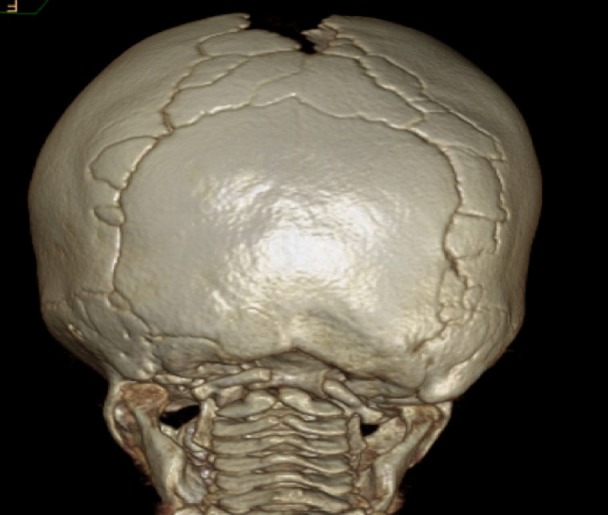
3D head computed tomography (CT) of patient with craniosynostosis Extreme example of sutural bones in the lambdoidal, sagittal, and asterion as seen in a patient with craniosynostosis (bilateral coronal synostosis) on 3D head CT.

Several clinical conditions causing skull deformity and suture strain have also been associated with increased incidence of wormian bones [8]. For example, in congenital hypothyroidism, the ossification centers of bone are affected. Mineralization, as mentioned previously, is an important factor in maintaining a fixed suture width in a growing cranium. A defect is this process may result in widened sutures – another feature associated with an increased number of wormian bones. Furthermore, patients with congenital hypothyroidism are more susceptible to brachycephaly deformities due to the growth delay at the base of their skulls [2] and decreased muscle tone. This decreased muscle tone, as postulated, leads to a less active baby and more time spent laying supine, compressing the occipital bone for extensive periods [8]. Another example of increased wormian bones can be seen in cleidocranial dysplasia, a condition in which a defect in the CFBA1 gene leads to the delayed closure of sutures, decreased ossification of cranial bones, and skull deformities such as frontal bossing and hyperteleorism [4]. Lastly, osteogenesis imperfecta also displays increased wormian bones. The defective collagen production in these patients result in widened skull sutures, decreased ossification of skull bones [8], and frontal bossing that can give the appearance of a triangular-shaped head [3]. Given that the most common location of wormian bones is within the lambdoid suture (also seen in our case report), this locational preference warrants further investigation (Figure 3). During development, sutures continue to grow until they reach their predetermined age for fusion, respective to their location. The lambdoid suture, joining the occipital bone with the two parietal bones superiorly, fuses during early childhood. We hypothesize that, as muscle groups such as the temporalis and trapezius grow and attach to the occipital and parietal bones, respectively, their development and contraction during this growth period may cause a widening of this suture, thereby increasing the chance for a wormian bone to develop within it.

Knowledge about wormian bones and their various possible locations are important to distinguish anatomical variants from pathologies such as fractures on imaging. Since these bones reside within sutures, they may give the appearance of a fractured segment or, if large enough, give the appearance of an accessory suture.

Instead of being a benign anatomical variant, wormian bones may negatively affect our health. In a radiographic study, wormian bones in the lambdoid suture were associated with a greater incidence of posterior parietal fractures in infants, and were thus postulated to weaken the skull [9]. Further studies are needed to examine the possible causal relationship and its implications. This information may help predict and prevent the risk of cranial fractures. In addition, with increasing reliance on medications to manage health issues, understanding the consequences of developing novel wormian bones may be a new concern. In a study by Mitalla et al. [10], the fetuses of rats fed aspirin on day 10 of their gestation had a 43% incidence of developing an accessory bone within the nasofrontal suture compared to the control group. This staggering association of a wormian bone developing in an extremely rare location at such a high incidence with medication use warrants further study. 

## Conclusions

Wormian bones are natural anatomical variants within a given population; thus, knowledge of their presence and various possible locations are important to distinguish them from pathologies. We reported a case of a sutural bone found between the nasal bone and frontal bones. To our knowledge, such an accessory bone in this location has not been previously reported. Several medical conditions and cultural practices can increase one's chances of developing wormian bones. Further studies are needed to elucidate other potential causal factors of wormian bones and their various health consequences.
